# Inoculation With *Azospirillum* spp. Acts as the Liming Source for Improving Growth and Nitrogen Use Efficiency of Potato

**DOI:** 10.3389/fpls.2022.929114

**Published:** 2022-07-28

**Authors:** Tahir Naqqash, Kauser Abdullah Malik, Asma Imran, Sohail Hameed, Muhammad Shahid, Muhammad Kashif Hanif, Afshan Majeed, Muhammad Javed Iqbal, Muther Mansoor Qaisrani, Jan Dirk van Elsas

**Affiliations:** ^1^Institute of Molecular Biology and Biotechnology, Bahauddin Zakariya University, Multan, Pakistan; ^2^National Institute for Biotechnology and Genetic Engineering (NIBGE), Faisalabad, Pakistan; ^3^Department of Biological Sciences, Forman Christian College, Lahore, Pakistan; ^4^Department of Biosciences, University of Wah Research Lab Complex, University of Wah, Wah, Pakistan; ^5^Department of Bioinformatics and Biotechnology, Government College University, Faisalabad, Pakistan; ^6^Department of Biological Sciences, University of Lahore, Sargodha Campus, Punjab, Pakistan; ^7^Department of Soil and Environmental Sciences, The University of Poonch Rawalakot, Rawalakot, Pakistan; ^8^Department of Bioinformatics, Khwaja Fareed University of Engineering and Information Technology, Rahim Yar Khan, Pakistan; ^9^Department of Microbial Ecology, Groningen Institute for Evolutionary Life Sciences, University of Groningen, Groningen, Netherlands

**Keywords:** plant growth regulator, *Azospirillum*, nitrogen fixation, potato tubers, *nif* H

## Abstract

Nitrogen (N) is one of the limiting factors for plant growth, and it is mainly supplied exogenously by fertilizer application. It is well documented that diazotrophic rhizobacteria improve plant growth by fixing atmospheric N in the soil. The present study investigates the nitrogen-fixing potential of two *Azospirillum* spp. strains using the ^15^N isotope-dilution method. The two diazotrophic strains (TN03 and TN09) native to the rhizosphere of potato belong to the genus *Azospirillum* (16S *rRNA* gene accession numbers LN833443 and LN833448, respectively). Both strains were able to grow on an N-free medium with N-fixation potential (138–143 nmol mg^−1^ protein h^−1^) and contained the nifH gene. Strain TN03 showed highest indole acetic acid (IAA) production (30.43 μg/mL), while TN09 showed highest phosphate solubilization activity (249.38 μg/mL) while both diazotrophs showed the production of organic acids. A ^15^N dilution experiment was conducted with different fertilizer inputs to evaluate the N-fixing potential of both diazotrophs in pots. The results showed that plant growth parameters and N contents increased significantly by the inoculations. Moreover, reduced ^15^N enrichment was found compared to uninoculated controls that received similar N fertilizer levels. This validates the occurrence of N-fixation through isotopic dilution. Strain TN09 showed higher N-fixing potential than TN03 and the uninoculated controls. Inoculation with either strain also showed a remarkable increase in plant growth under field conditions. Thus, there were remarkable increases in N use efficiency, N uptake and N utilization levels. Confocal laser scanning and transmission electron microscopy showed that TN03 is an ectophyte, i.e., present outside root cells or within the grooves of root hairs, while TN09 is an endophyte, i.e., present within root cells, forming a strong association withroot it. This study confirms that diazotrophic *Azospirillum* spp. added to potato systems can improve plant growth and N use efficiency, opening avenues for improvement of potato crop growth with reduced input of N fertilizer.

## Introduction

An increase in crop production to fulfill consumer demand and provide food for the world's growing population involves the substantial use of pesticides and chemical fertilizers, which are often overused in the soil (Singh et al., 2020). Chemical fertilizers, particularly sources of nitrogen (N), crop yield by 50% on average compared to yield without their use. However, the dependency of the current agricultural system on chemical fertilizers has been alarming because the indiscriminate use of the latter has resulted in major environmental issues such as air pollution, water eutrophication, and soil acidification (Dungait et al., 2012). Approximately 235 million tons of N fertilizers are being produced each year industrially using fossil fuels (Ghavam et al., 2021). Without N fertilizers, it is estimated that the global production of food would be insufficient to feed the world's current population (Connor, 2008). However, adding nitrogen fertilizer to increase agricultural yields, might have hit a plateau. Excessive nitrogen fertilizer results in reduced nitrogen use efficiency (NUE) because of the rapid loss of N from denitrification, ammonia volatilization, leaching, and surface runoff in the soil-water system (Smil, 1999).

Two effective ways have been suggested to minimize the dependency on N fertilization, including; i) developing plants with enhanced NUE and ii) use of diazotrophic microbes to promote nitrogen fixation (Ladha et al., 2016). These diazotrophic microbes help plants in nutrient uptake by acting as phyto-stimulators, biofertilizers, and alleviators of environmental stressors (Pii et al., 2015). The most important genera of diazotrophic microbes associated with agriculturally important plants are *Azotobacter, Arthrobacter, Azospirillum, Burkholderia, Bacillus, Clostridium, Herbaspirillum, Pseudomonas*, and *Gluconacetobacter* (Naqqash et al., 2016b; Zeffa et al., 2019; Chea et al., 2021).

With 19 different species, *Azospirillum* is one of the best-studied genera of plant growth-promoting rhizobacteria (PGPR) (Cassán and Diaz-Zorita, 2016). Different species of *Azospirillum* such as *A. lipoferum, A. brasilense, A. oryzae*, and *A. halopraeferens*, can promote plant growth through various mechanisms, including the release and biosynthesis of indol-3-acetic acid, amino acids, gibberellins, polyamines, and cytokinins, which favor root growth and subsequently increase nutrient and water uptake in the plant (Gupta et al., 2015). Apart from from these traits, the nitrogen-fixing ability of *Azospirillum* spp. contributes directly to making atmospheric N accessible to plants (Pankievicz et al., 2015; De-Bashan et al., 2016). Azospirillum, like other PGPR, promotes the assimilation of N-compounds in plants and favors a reduction of mineral fertilizer application and N_2_O release, with a substantial increase in crop production (Bashan, 1998; Martins Da Costa et al., 2017). Multiple mechanisms are synergistically involved in the plant growth-promoting potential of *Azospirillum* sp., as explained in Bashan and Levanony's “Multiple Mechanism Theory,” (Bashan and De-Bashan, 2010).

*Solanum tuberosum* L. (potato) is the most common non-grain staple diet in many regions of the world (Calvo et al., 2010; Hanif et al., 2015a). It provides maximum dry matter per unit time and area compared to cereal crops (Ezekiel et al., 2013), this is one of the main reasons scientists are interested in its use, ensuring future food security. However, potato needs a lot of fertilizer, which has a bearing on the production costs, given that fertilizer prices keep on increasing. Excessive use of fertilizer also affects the ecosystem. In fact, fertilizers are the primary source of phosphorus (P) and N pollution in water and soil. Potato requires 180 to 240 kg N ha^−1^ fertilizer to yield maximum production (35 to 45 tons ha^−1^). Usually, plants take up only 40 to 50% while the remaining N is released into the environment. The current study was conducted to examine the nitrogen-fixation potential of two novel *Azospirillum* spp. using a ^15^N isotope dilution approach. We further examined their practical usefulness with respect to potato growth and yield, as evaluated under both controlled and field conditions.

## Materials and Methods

### Soil Sampling and Bacterial Isolation

Soil samples from the rhizosphere of potato plants were collected from two different fields located in Gujranwala (74° 7.40 E, 32° 03.324 N) and Jhang (72° 16.08 E, 31° 13.712 N). Physiochemical parameters of soil such as pH, texture, electrical conductivity (EC), organic matter, total N, total P, total potassium (K), total mineral N and saturation were analyzed following standard methods (Naqqash et al., 2020).

Rhizobacteria were isolated from the soil (1 g) on LB agar plates using the serial dilution technique (Somasegaran and Hoben, 2012). The rhizospheric soil was mixed with the 0.89% (w/v) saline solution followed by serial dilution. The colony-forming units (CFU/g) of the general rhizobacterial population were counted by spreading 100 μL of dilution on the LB agar plates following incubation for 2–7 days at 28 ± 2°C. For isolation of nitrogen-fixing rhizobacteria, 0.1 g soil containing roots were mixed with nitrogen-free malate (NFM) semi-solid media in 1.5 ml Eppendorf tubes following incubation at 28 ± 2°C (Okon et al., 1977). The culture was enriched and further streaked on LB and NFM agar plates. Single colonies showing different morphological characteristics were purified and two rhizobacterial morphotypes TN03 and TN09 isolated on both agar plates (LB and NFM) were selected for further characterization and analysis in detail.

### Morphological Characterization

For investigating colony and cell morphology, rhizobacterial isolates (TN03 and TN09) were grown on LB agar plates at 28 ± 2°C for 24 h. The motility and shape of selected rhizobacterial isolates were observed using a light microscope. Gram's reaction was performed according to Vincent's method (Vincent, 1970).

### Phenotypic Microarrays

BIOLOG-GN2 microplates were used to determine the metabolic potential of rhizobacterial isolates (TN03 and TN09) (Muller and Ehlers, 2005). Both strains were grown for 48 h on LB agar plates at 28 ± 2°C. The freshly grown culture was mixed with DEPC water (1 ml) in Eppendorf tubes following starvation for 3 h. This culture was further mixed with redox indicator and IF-0a inoculation fluid as suggested by the manufacturer. In the carbon utilization plate (PM2A), 100 μl of this mixture was added following incubation for 24 h at 28 ± 2°C (Biolog, Hayward, CA) and were analyzed using the VERSA max micro-plate reader (Molecular Devices, USA) having soft-max software.

### Nitrogen Fixation

The acetylene reduction assay (ARA) was used for determining the nitrogen-fixing potential of bacteria (Hardy et al., 1968). Both isolates were grown at 28 ± 2°C for 3 days on NFM-semisolid media and analyzed for nitrogenase activity using a gas chromatograph equipped with a flame ionization detector and a Porapak N-column (Thermoquest, Trace-GC, Rodon Milan, Model K, Italy). The ARA activity of TN03 and TN09 was measured in moles of ethylene mg^−1^ protein h^−1^ (Park et al., 2005). Bradford's method (Bradford, 1976) was used for the determination of protein concentration.

### Indole-Acetic Acid (IAA) Production

Single colonies of freshly grown bacteria were inoculated into the LB broth (100 ml) with or without L-tryptophan (100 mg L^−1^) following incubation for 2 days at 28 ± 2°C with continuous shaking. After centrifugation for 10 min at 13,000 g, the 100 μL supernatant was collected and mixed with Salkowski's reagent (100 μl) following incubation for 30 min at room temperature. The supernatant was further acidified at pH=2.8 and extracted using the same quantity of ethyl acetate for quantification. The extract was then evaporated until it dried completely and was collected in methanol following filtration using a nylon filter (0.2 μm). High-performance liquid chromatography (HPLC) (Perkin Elmer, USA) was used to analyze purified extract, equipped with a C-18 column. The mobile phase used was acetic acid, methanol, and water (1:30:70 v/v/v) at a 1 ml/min flow rate (Tien et al., 1979).

### P-Solubilization and Organic Acid Production

The Pikovskaya's agar plates were spot-inoculated with pre-grown rhizobacterial strains and incubated for 5–7 days at 28 ± 2°C (Pikovskaya, 1948). The halo zone formation around the rhizobacterial colonies showed the potential of isolates to solubilize tri-calcium phosphate. Rhizobacteria were inoculated in triplicate in Pikovskaya's broth (100 ml) and cultured in an orbital shaker at 150 rpm for 12 days at 28 ± 2°C for quantification. At 3, 5, 7, and 12 days, 20 ml culture was taken, and cell-free supernatant was recovered by centrifugation for 10 min at 13,000 × g. The Phospho-molybdate blue color technique was used to determine P-solubilization (Murphy and Riley, 1962). The cell-free supernatant was filtered and examined on HPLC at 210 nm containing Turbochrom software. The C-18 column was used as the mobile phase as described above against the standards of organic acids: lactic, gluconic, oxalic, ascorbic, tartaric, and malic acid (Shahid et al., 2012).

### Amplification and Sequence Analysis of 16S *rRNA* and *nif*H Gene

A genomic DNA extraction kit (Invitrogen) was used to extract the genomic DNA of bacteria. The universal primers (968F and 1406R) were used to amplify the 16S *rRNA* gene under the following conditions: initial denaturation at 95°C for 5 min followed by denaturation at 94°C for 60 s, annealing at 56°C for 60 s, extension at 72°C for 120 s and final extension at 72°C for 10 min for 30 cycles as described previously (Naqqash et al., 2020). For *nif* H gene amplification, nested PCR was performed using FGPH19 (Simonet et al., 1991) and PolR (Poly et al., 2001) primers for 1st PCR that resulted in 490 bp product while PolF and AQER (Poly et al., 2001) primers were used for 2nd PCR using the product of 1st PCR as a template. The products of 16S *rRNA* and *nif* H gene were analyzed on ethidium bromide-stained agarose gel (1%). Amplified PCR products of 16S *rRNA* and *nif* H gene were sequenced from Macrogen (South Korea) and sequences were submitted to the NCBI GenBank. The sequences were analyzed by Sequence Scanner Software while MEGA6 Software was used for determining phylogeny. Maximum likelihood (ML) was used to perform phylogenetic analysis, and the bootstrap value of 70% or above was maintained to indicate well-supported nodes (Hillis and Bull, 1993).

### Plant Inoculation Studies

#### Pot Experiment

A pot experiment was conducted for estimating the N-fixing potential of isolates and their impact on potato growth. The medium-sized (2–3 cm) potato tubers were surface-sterilized for 8–10 min using 10% sodium hypochlorite followed by washing with distilled water. Isolates were grown for 24 h at 28 ± 2°C in LB broth (100 mL) and their cells were collected through centrifugation for 15 min at 4,000 × g at room temperature followed by resuspension in 0.89% saline solution. A single potato tuber having two eye buds was placed in each inoculum (10^8^ CFU/ml) individually for 20 min. Two different experiments were conducted under complete randomized design (CRD) to evaluate the % N-fixation potential of diazotrophs and their role in improving N contents, biomass and growth of potato tubers. In 1st experiment, the growth-promoting potential and nitrogen use efficiency were evaluated in sterilized sand, while in the 2nd experiment, pots were filled with sterilized sandy loamy soil (8 kg) having 8.1 pH, 0.6% OM, 0.06% total N, and 2.5 dSm^−1^ EC. The per cent nitrogen fixation (%Ndfa) was estimated using the ^15^N-natural abundance approach and ^15^N-dilution technique. In 1st experiment, there were four treatments: T_1_ = Un-inoculated + N^0^ (-ve control treatment; without N), T_2_ = Un-inoculated + N^F^ (+ve control treatment; recommended full dose of 250 Kg ha^−1^ of N), T_3_ = Inoculated with strain TN03 + N^0^ and T_4_ = Inoculated with strain TN09 + N^0^, each replicated four times. In the 2nd experiment, there were nine treatments: T1 = Un-inoculated + ^15^N^0^ (−ve control treatment), T_2_ = Inoculated with strain TN03 + ^15^N^0^, T_3_ = Inoculated with strain TN09 + ^15^N^0^, T_4_ = Un-inoculated + ^15^N^1/2^ (+ve control treatment; 175 Kg ha^−1^), T_5_ = Inoculated with strain TN03 + ^15^N^1/2^, T_6_ = Inoculated with strain TN09 + ^15^N^1/2^, T_7_ = Un-inoculated + ^15^N^F^ (++ve control treatment; 250 Kg ha^−1^), T_8_ = Inoculated with strain TN03 + ^15^N^F^, and T_9_ = Inoculated with strain TN09 + ^15^N^F^, each replicated four times. ^15^N was administered in the form of 5% ammonium sulfate a.e (atomic access). Plants were grown in the growth room (20 ± 8°C day/night temperature and 16/8 h light/dark photoperiod) and were irrigated with distilled water and Hoagland solution without N. The second bacterial inoculation was done after the emergence of seedlings (15 days) by mixing them with Hoagland solution. Sixty days after sowing, plants were harvested and processed to produce data regarding N content and plant growth.

##### Estimation of Total N

The Kjeldahl method was used for determining total N in all treated and non-treated plants. For this, plants were ground in a stainless-steel grinding mill after drying them for 48 h at 60°C. The 2 g powder was mixed with concentrated H_2_SO_4_ (5 ml) and digestion mixture [K_2_SO_4_ (100): CuSO_4_ (10): Se (1)] in a digester (Digester 1016, Tecator). The NaOH solution was used for steam distillation of this digested solution. The ammonia produced during this process was precipitated using boric acid (2%) solution. The total N was calculated by titration of the ammonium-boric acid mixture against a standard solution of H_2_SO_4_ solution using the following equation.

N (mg g^−1^) = Blank (acid used) × 14 × Acid normality used for titration ÷ weight of the sample.

##### Estimation of ^15^ N Abundance

After estimating total N, the titrated solution was acidified by mixing the few drops of H_2_SO_4_. Samples were made concentrated (5 ml) by evaporating water and tested for ^15^N abundance using the mass spectrometer equipped with a double-inlet system following the Rittenburg technique. The ^15^N was released using sodium hypobromite (Hauck, 1982). The N-fixation rate was determined as a percentage of ^15^N a.e (atomic excess) using the following equation Malik et al. (1987).


$$\begin{array}{r} \begin{array}{c} {\;}^{15}\text{Nisotopedilutionmethod}:\\ \text{\%Ndfa}=\left(1-{\text{\%}}^{15}{\text{N}}_{\text{FS}}/{\text{\%}}^{15}{\text{N}}_{\text{NFS}}\right)\times 100\\ \text{Naturalabundance}:\\ \text{\%Ndfa}=\left({\text{δ}}^{15}{\text{N}}_{\text{NFS}}{-}^{15}{\text{N}}_{\text{FS}}/{\text{δ}}^{15}{\text{N}}_{\text{NFS}}\right)\times \;100 \end{array} \end{array}$$

Where

%Ndfa = % nitrogen fixation,

δ15N = natural abundance of 15N,

FS = fixing system,

NFS = non fixing system.

#### Field Experiment

Both diazotrophs were further investigated for their growth promotion and NUE in field conditions. Randomized-complete block design (RCBD) experiment was conducted using the “Kuroda” potato cultivar, at two different locations i.e., Field 1: National Institute for Biotechnology and Genetic Engineering (NIBGE), Faisalabad and Field 2: Potato Research Institute (PRI), Sahiwal. Before sowing, the data regarding bacterial population and physiochemical properties of soil was taken as described above. The experiment was consisted of five different treatments including T_1_ = Un-inoculated + N^0^ (−ve control treatment), T_2_ = Un-inoculated + N^1/2^ (+ve control treatment; 175 Kg ha^−1^), T_3_ = Un-inoculated + N^F^ (++ve control treatment; 250 Kg ha^−1^), T_4_ = Inoculated with strain TN03 + N^1/2^ and T_5_ = Inoculated with strain TN09 + N^1/2^, each with four replicates. Before sowing, the field was properly irrigated to ensure that there was enough moisture for tuber germination. The soil of both fields was cultivated by plowing (3–4 times) following planking to prepare the field. For inoculation, potato tubers were soaked in each bacterial inoculum (TN03 and TN09) for half an hour before sowing. On ridges, manual sowing was done. The size of each plot was 3.6 × 5.25 m (18.9 m^2^), with 75 cm R × R distance and 30 cm P × P distance. Nitrogen was administered (as urea) in two halves, the first half at the sowing time and the second half at the time of first irrigation, via broadcasting. Furthermore, at the sowing time, potassium minerals (as potash muriate) and phosphorous (as single-super phosphate) were also applied (150 kg ha^−1^ each). For all five treatments, all the other plant protection measures (disease control, insect, weed, and pest) and agronomic methods were kept the same. After 60 days of sowing, plants were harvested and different agronomic parameters were measured such as Plant height (cm), Number of branches plant^−1^, Number of compound leaves plant^−1^, Number of tubers plant^−1^, Plant fresh weight (g), Plant dry weight (g), Tuber fresh weight (g), Tuber dry weight (g), Number of tubers per plant, Tuber yield (Kg ha^−1^) and Plant N contents (mg g^−1^). NUE, nitrogen uptake (NUP), and N utilization (NUT) were calculated following the method described previously (Moll et al., 1982).

NUT (tones ha^−1^ tubers/mg g^−1^ N accumulated) = [tuber yield (tones ha^−1^) with fertilizer (kg ha^−1^) - tuber yield without fertilizer] ÷ [total N accumulated (mg g^−1^) in tubers with fertilizer (kg ha^−1^) - total N accumulated (mg g^−1^) in tubers without fertilizer (kg ha^−1^)]

NUP (mg g^−1^ N accumulated/kg ha^−1^ N applied) = [total N accumulated (mg g^−1^) in tubers with fertilizer (kg ha^−1^) - total N accumulated (mg g^−1^) in tubers without fertilizer (kg ha^−1^)] ÷ Rate of N fertilizer applied (kg ha^−1^)

NUE (tones ha^−1^ tubers/ kg ha^−1^ N applied) = [tuber yield (tones ha^−1^) with fertilizer (kg ha^−1^) - tuber yield without fertilizer] ÷ Rate of N fertilizer applied (kg ha^−1^).

### Root Colonization Studies

#### Ultrastructure Analysis of Inoculated Potato Roots

At the ultrastructure level, root colonization was investigated using Transmission Electron Microscope (TEM). The roots of potato plants were uprooted carefully; cut into small pieces (1–3 cm) followed by washing with distilled water. Water agar was used to embed the agar cubes (2–3 mm^3^). Dehydration, polymerization, and sample fixation in glutaraldehyde (5%) were performed as previously reported (Naqqash et al., 2016b). For the root colonization study, ultrathin sections were cut using an ultra-microtome (RMC-700) and were examined using TEM (JEOL, USA).

#### Confocal Microscopy Analysis for Root Colonization

Bacteria were transformed using the PBBRMCS-1plasmid, which encodes *amp*R and contains a yellow fluorescent protein (*yfp*) gene (Shahid et al., 2012). LB agar plates supplemented with 50 μg/ml ampicillin were used for screening of transformants and validated at 480 nm using a confocal laser scanning microscope (CLSM). For inoculation, *yfp*-transformed strains were grown in LB-ampicillin media (10^8^ CFU ml^−1^). Cells were harvested by centrifugation at 8,000 × g following resuspension in 0.89% saline. Surface sterilized medium-sized potato tubers were inoculated with *yfp*-transformed strains and were grown under controlled conditions (as described above) in the sand for 30 days, with four replicates along with un-inoculated control. Colonization of 30-day old inoculated potato roots was observed under CLSM (Olympus Fluo-view Ver.1.3) following the procedure described previously (Naqqash et al., 2016b).

### Statistical Analyses

The data of the pot experiment was statistically analyzed by the analysis of variance (ANOVA) approach using Statistix 8.1 software. The least significant difference (LSD) test at the 5% probability was used for comparing the differences between means of different treatments. The data of field experiments were analyzed by principal component analysis (PCA) and a heat map was generated to identify grouping patterns of different treatments, locations, and growth parameters using the Origin Pro 2021 Software.

## Results

### Identification and Characterization of Soils and Diazotrophic Strains

The soil samples collected from the potato growing regions in Gujranwala and Jhang showed loamy and sandy loam textures, respectively with a slight change in total N (%) and pH, while soil EC ranged from 1.2 to 2.3. The organic matter (%) and total P in the Jhang soil sample were substantially higher than in the Gujranwala soil sample. Gujranwala soil showed levels of total mineral N, phosphorous and saturation exceeding those of Jhang soil. The physiochemical properties of both soil samples are listed in Table 1.

**Table 1:** Physiochemical characteristics of Gujranwala and Jhang soil samples collected from potato rhizosphere.

**Physiochemical characteristics**	**Gujranwala**	**Jhang**
Soil pH	8.2	8.4
Soil texture	Loam	Sandy Loam
EC (d S m^−1^)	2.3	1.2
Organic matter (%)	1.15	1.32
Total N (%)	0.09	0.08
Total P (mg Kg^−1^)	96.3	75.6
Total K (mg Kg^−1^)	140	560
Total mineral N (mg kg^−1^)	8.11	6.13
Saturation (%)	34	26

Both diazotrophic strains formed small, round, white colonies on LB agar plates, were vibroid-shaped and showed Gram-negative reactions (Table 2). Sequence analysis of the 16S *rRNA* gene showed both isolates belonged to the genus *Azospirillum*. Isolate TN03 (Accession No. LN833443) showed 99% similarity with *Azospirillum* sp. YM 249, while TN09 (Accession No. LN833448) showed 99% similarity with *Azospirillum brasilense* strain Gr 22 (Table 2 and Supplementary Figure 1). In the analysis, A. *halopraeferens* (NR_044859) was used as the root. Phylogenetic analysis of TN03 and TN09 in the GenBank database further validated their evolutionary relationships with the indicated closely.

**Table 2:** Morphological, biochemical and molecular identification of TN03 and TN09 strains purified from potato rhizosphere.

**Identification parameters**	**TN03**	**TN09**
Isolation site	Gujranwala	Jhang
Colony morphology	Small, round and white	Small, round and white
Cell morphology	Vibroid	Vibroid
Gram's reaction	–ve	–ve
ARA (nmoles mg^−1^ protein h^−1^)	138.21 ± 15.21	143.07 ± 11.98
IAA (μg ml^−1^)	30.43 ± 3.16	19.77 ± 1.45
GenBank Accession No. (*16S rRNA*)	LN833443	LN833448
GenBank match for *16S rRNA* (%)	*Azospirillum* sp. YM 249 (99%)	*Azospirillum brasilense* strain Gr22 (99%)
GenBank match for *nif*H sequence (%)	*Azospirillum brasilense* strain Gr37 (99%)	*Azospirillum brasilense* strain Gr42 (100%)
Accession No. (*nif*H sequence)	LT596586	LT596588

Analysis of the *nif* H gene sequence showed that both isolates (TN03 and TN09) had 99–100% similarity with similar sequences of different strains of *Azospirillum brasilense*. Briefly, the sequence of TN03 (Accession No. LT596586) showed 99% similarity with the *A. brasilense* (X51500.1) *nif* H gene, while the sequence of TN09 (Accession No. LT596588) showed similarity with the similar sequence of *A. formosense* (HM193519.1; Supplementary Figure 2; Table 2). The presence of *the nif* H gene indicated that both isolates have the potential for N-fixation which was further validated by testing their acetylene reduction activities (ARA). Isolate *Azospirillum* sp. TN09 showed the highest highest ARA (143.07 nmol mg^−1^ protein h^−1^), while TN03 showed slightly lower (138.21 nmol mg^−1^ protein h^−1^) N-fixation potential (Table 2).

**Figure 1:**
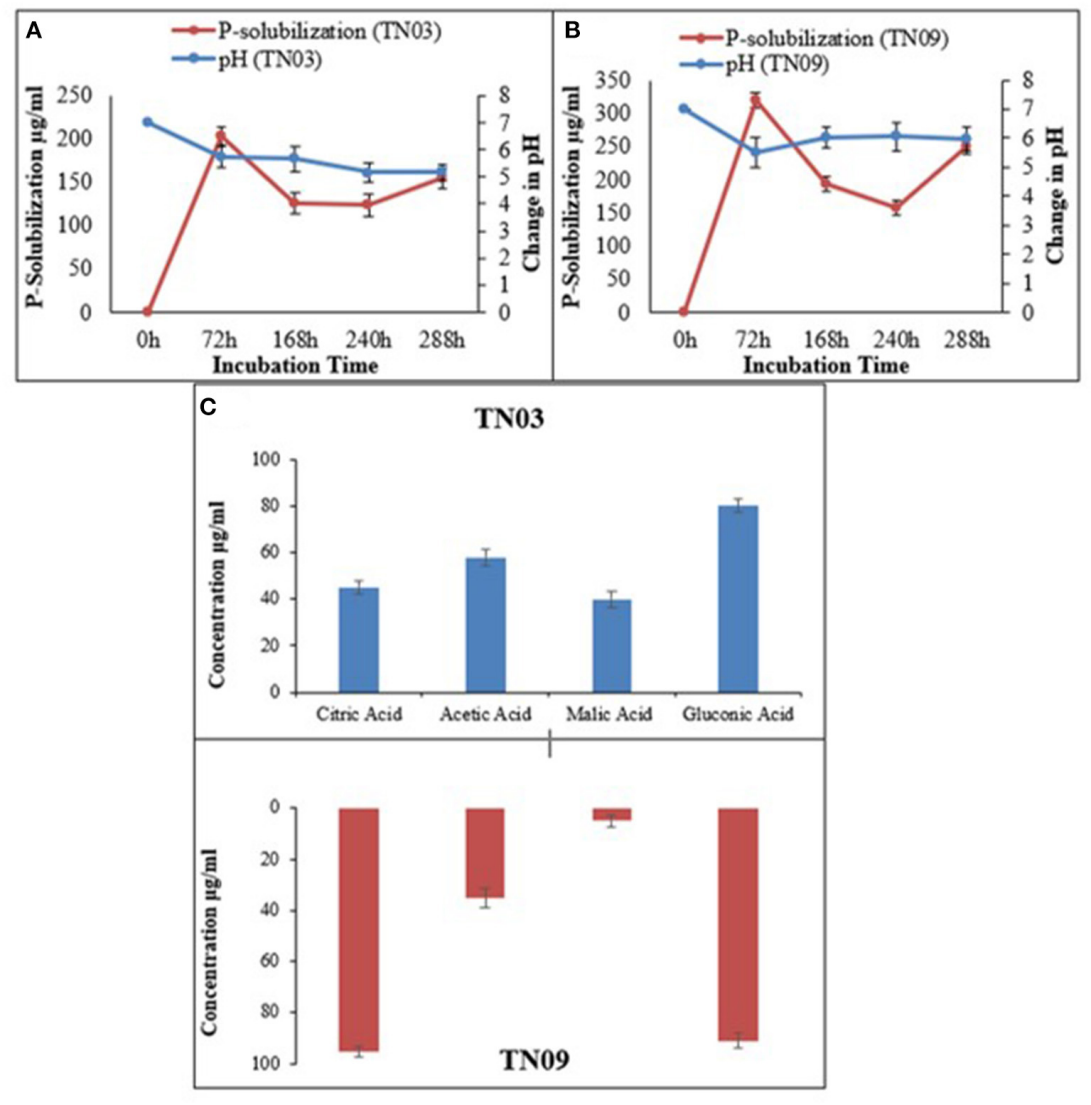
Relationship between phosphate solubilization and pH change of *Azospirillum* spp. strains **(A)** TN03; **(B)** TN09; in Pikovskaya's media; and **(C)** organic acid production.

Both strains showed limited metabolic potential i.e., they utilized only 9 and 10 carbon sources, respectively of the BIOLOG panel (Supplementary Table 1). Both showed potential for IAA production, as well as P-solubilization (as shown by the halo-zones on Pikovskaya's agar plates supplemented with tricalcium phosphate; Figures 1A,B). *Azospirillum* sp. TN03 showed maximum IAA production (30.43 μg/ml) (Table 2), while TN09 showed higher P-solubilization (249.38 μg/ml) potential with a constant decrease in pH (5.98) (Figure 1B). Both strains showed the production of citric acid, acetic acid, malic acid, and gluconic acid (Figure 1C). Strain TN03 showed maximum production of acetic acid (58 μg/ml ± 3.47) and malic acid (40 μg/ml ± 3.37), while TN09 showed the highest production of citric acid (95 μg/ml ± 2.01) and gluconic acid (91 μg/ml ± 2.94).

### Rhizosphere Survival and *in vivo* N-Fixation Potential Using the ^15^N Technique

**Figure 2:**
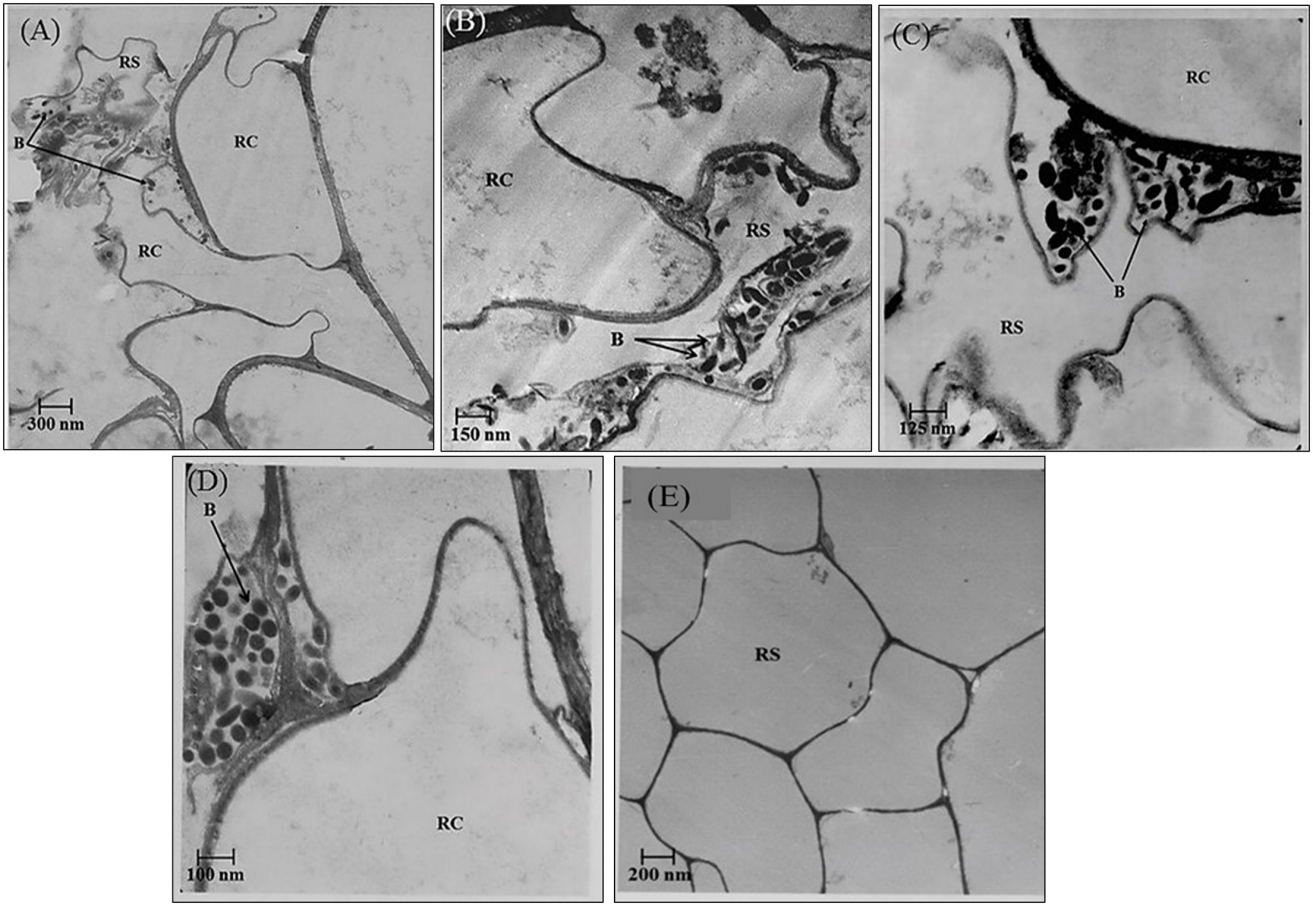
Electron-micrograph of ultrathin sections of root inoculated with *Azospirillum* sp. TN03 after 30 days. RC = Root cell, B = Bacterium and RS = Rhizosphere. *Azospririllum* sp. TN03 formed micro-colonies over the root cells surface and in the root hairs grooves **(A)**. Bacterial cell clusters are present on the root epidermal cells **(B–D)** forming multiple layers of cell on it and in uninoculated control plants no microbial cells or clusters were observed **(E)**.

**Figure 3:**
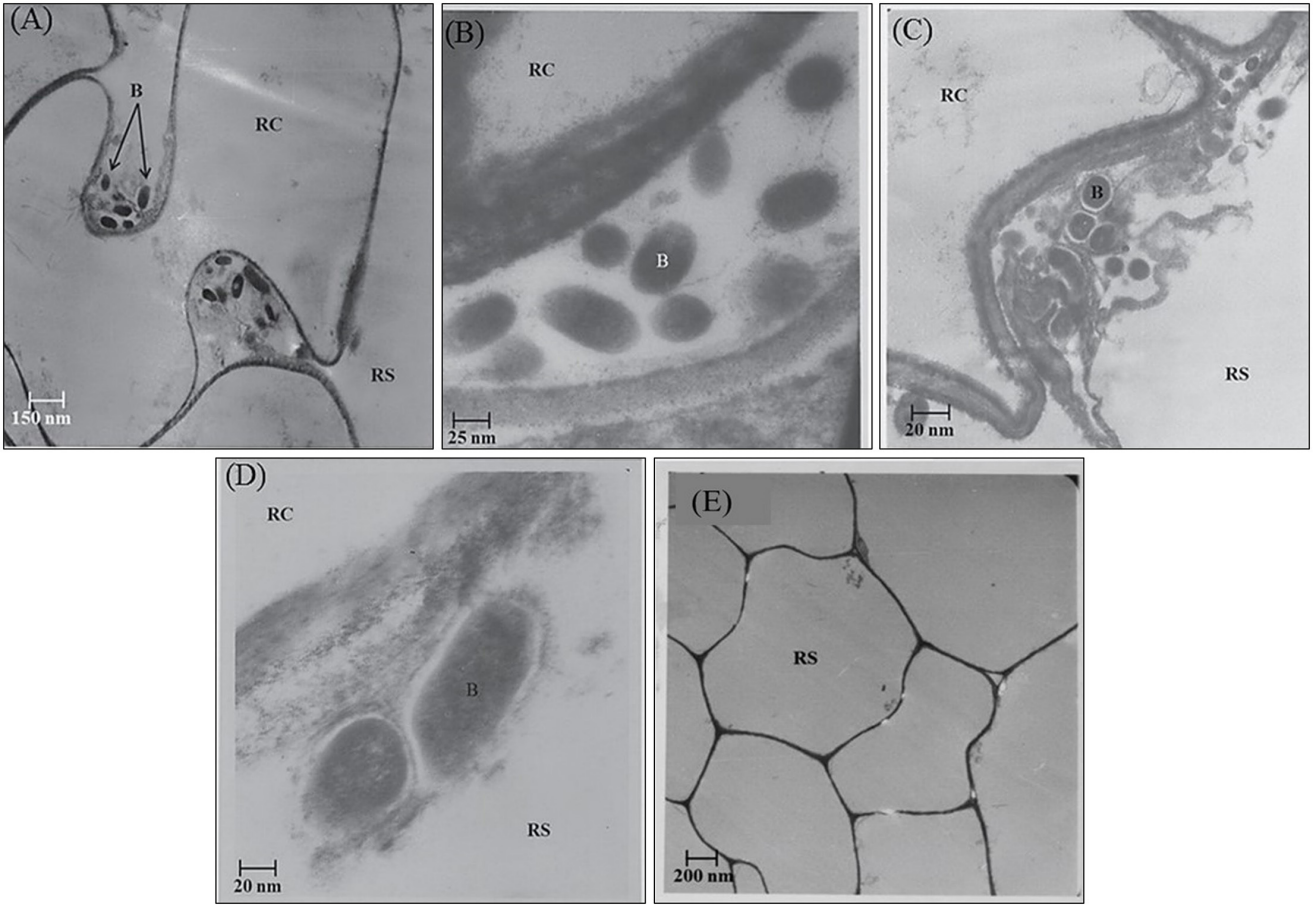
Electron-micrograph of ultrathin sections of root inoculated with *Azospirillum* sp. TN09 after 30 days. RC = Root cell, B = Bacterium and RS = Rhizosphere. Cells of *Azospirillum* sp. TN09 form micro-colonies among root cells forming clusters of bacterial cells **(A,B)**. These multicellular aggregates on root surface were also embedded within the extracellular matrix showing strong adhesion with the roots of potato plants **(C,D)** and in uninoculated control plants no microbial cells or clusters were observed **(E)**.

**Figure 4:**
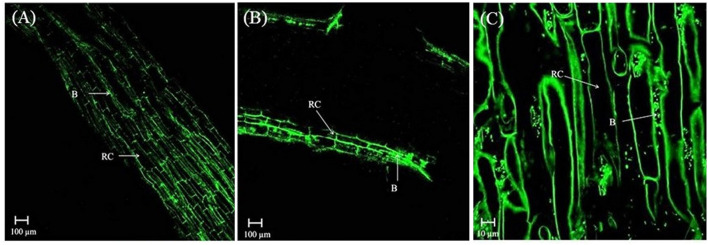
Confocal microscopic image of potato root inoculated with YFP-labeled *Azospirillum* sp. TN03 in sterilized sand after 25 days. Lateral and primary roots of potato are ideal colonization sites of *Azospirillum* sp. TN03 **(A,B)**. Bacterial aggregates forming macro-colonies were observed on root epidermal cells validating association of TN03 strain with potato roots **(C)**. RC, Root cell; B, Bacterium.

**Figure 5:**
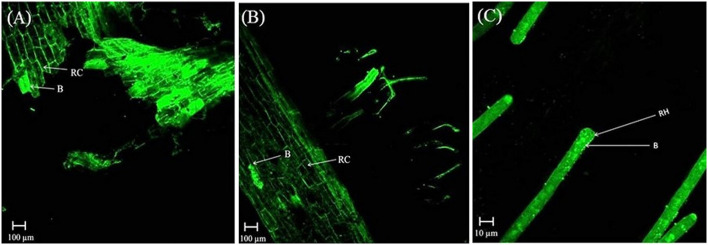
Confocal microscopic image of potato root inoculated with YFP-labeled *Azospirillum* sp. TN09 in sterilized sand after 25 days. Bacterial cells forming thick macro-colonies of *Azospirillum* sp. TN09 were observed over the potato root surface **(A,B)**. Root lateral area and hair tips were the preferred sites of *Azospirillum* sp. TN09 colonization shows the association of TN09 strain with potato roots **(C)**. RC, Root cell; RH, Rhizosphere; B, Bacterium.

In the rhizosphere of potato, the population densities of TN03 and TN09 apparently depended on the plant growth stage. After 20 days of inoculation, strain TN09 maintained a larger population density (7.21 log CFU/g) than *Azospirillum* sp. TN03 (6.84 log CFU/g). The population densities of both strains had decreased (to 5.87 and 6.35 log CFU/g) after 60 days of inoculation (Supplementary Figure 3). Ultrastructure studies further validated the root colonization potential of both strains. Using TEM, the inoculated roots showed that strain TN03 inhabited the rhizoplane by forming microcolonies outside the root cells. The bacterial cells resided in grooves formed by root hairs and colonized intercellular spaces on the root cell surface by forming macrocolonies, showing close association with the plant cell wall (Figures 2A–D). Strain TN09 inhabited the spaces among the root cells by forming micro-colonies. Multicellular aggregates of this bacterium were present in the extracellular matrices close to the cell wall of the roots, indicating a strong association of this bacterium with the potato roots (Figures 3A–D). Uninoculated control plants showed no bacterial cells in their roots or rhizospheric regions (Figures 2E, 3E). The colonization potential of both strains was further validated using confocal microscopy. After 25 days of inoculation, the root of the potato plants was covered with YFP-labeled *Azospirillum* sp. TN03, showing that lateral and primary roots are preferred colonization sites for this bacterium. Moreover, bacterial aggregates were also observed on the epidermal root cells (Figure 4). In potato plants inoculated with YFP-labeled *Azospirillum* sp. TN09, the primary root hair tips and lateral tips were the preferred colonization sites. Bacterial aggregates, in the form of thick macrocolonies, were observed on the entire surface of the potato plant roots (Figure 5).

The potential of both *Azospirillum* spp. to promote plant growth was investigated by inoculating potato tubers in pots. The results showed a positive impact for both isolates regarding the N contents and biomass of the inoculated potato plants. The effect of strain TN03 was significant with respect to shoot and root length while the effect of TN09 was more prominent with respect to the fresh and dry weight of shoot and root (Table 3). Remarkably, the increase in N content of shoot and root was the same for both inoculants (Table 3).

**Table 3:** Inoculation response of *Azospirillum* spp. strains TN03 and TN09 of 1st pot experiment and 2nd pot experiment on root and shoot.

	**Experiment 1**			
	**Shoot Length (cm)**	**Shoot Fresh weight (g)**	**Shoot Dry weight (g)**	**Shoot N (mg/g)**
T1	10.50 ± 0.75 C	24.75 ± 2.78 C	3.00 ± 0.62 B	3.07 ± 0.12 B
T2	22.27 ± 1.82 A	54.35 ± 4.41 A	5.35 ± 0.68 A	4.20 ± 0.42 A
T3	16.37 ± 1.09 B	46.02 ± 3.03 B	4.37 ± 0.45 A	4.04 ± 0.58 A
T4	12.35 ± 1.03 C	50.52 ± 3.56 AB	4.65 ± 0.58 A	4.09 ± 0.47 A
	**Root Length (cm)**	**Root Fresh weight (g)**	**Root Dry weight (g)**	**Root N (mg/g)**
T1	10.42 ± 0.91 C	19.55 ± 1.75 B	1.77 ± 0.30 B	1.26 ± 0.11 B
T2	25.75 ± 2.29 A	45.67 ± 3.17 A	4.65 ± 0.86 A	2.40 ± 0.09 A
T3	14.52 ± 1.10 B	40.97 ± 3.43 A	3.85 ± 0.59 A	2.35 ± 0.07 A
T4	12.70 ± 0.98 BC	43.32 ± 3.13 A	3.95 ± 0.83 A	2.39 ± 0.10 A
	**Experiment 2**			
	**Shoot Length (cm)**	**Shoot Dry weight (g)**	**Root Length (cm)**	**Root Dry weight (g)**
T1	16.46 ± 0.61 E	3.63 ± 0.50 E	11.47 ± 1.23 D	2.53 ± 0.35 D
T2	17.60 ± 1.21 DE	4.16 ± 0.35 D	12.87 ± 1.08 CD	2.86 ± 0.40 CD
T3	17.53 ± 0.97 E	3.96 ± 0.40 D	15.21 ± 1.15 C	3.26 ± 0.45 C
T4	17.60 ± 1.21 DE	4.90 ± 0.45 C	16.09 ± 1.17 C	3.03 ± 0.25 CD
T5	18.46 ± 0.86 DE	5.76 ± 0.45 B	15.69 ± 1.32 C	4.23 ± 0.30 B
T6	19.46 ± 0.86 CD	5.96 ± 0.32 B	21.04 ± 1.15 C	4.43 ± 0.25 B
T7	23.83 ± 1.20 A	7.80 ± 0.46 A	23.98 ± 1.94 AB	5.33 ± 0.45 A
T8	21.30 ± 1.05 BC	7.26 ± 0.50 A	26.25 ± 2.47 A	5.36 ± 0.45 A
T9	22.63 ± 1.35 AB	7.73 ± 0.35 A	27.28 ± 3.60 A	5.50 ± 0.36 A

*Letters A, B, C, and D represent a significant difference between the means of all these treatments. Values are mean ± Standard Deviation. Number of biological replicates=4*.

*1st pot experiment: T_1_ = Un-inoculated + N^0^ (-ve control treatment; without N), T_2_ = Un-inoculated + N^F^ (+ve control treatment; recommended full dose of N), T_3_ = Inoculated with strain TN03 + N^0^ and T_4_ = Inoculated with strain TN09 + N^0^; 2^nd^ pot experiment: T_1_ = Un-inoculated + ^15^N^0^ (-ve control treatment), T_2_ = Inoculated with strain TN03 + ^15^N^0^, T_3_ = Inoculated with strain TN09 + ^15^N^0^, T_4_ = Un-inoculated + ^15^N^1/2^ (+ve control treatment), T_5_ = Inoculated with strain TN03 + ^15^N^1/2^, T_6_ = Inoculated with strain TN09 + ^15^N^1/2^, T_7_ = Un-inoculated + ^15^N^F^ (++ve control treatment), T_8_ = Inoculated with strain TN03 + ^15^N^F^, and T_9_ = Inoculated with strain TN09 + ^15^N^F^*.

**Figure 6:**
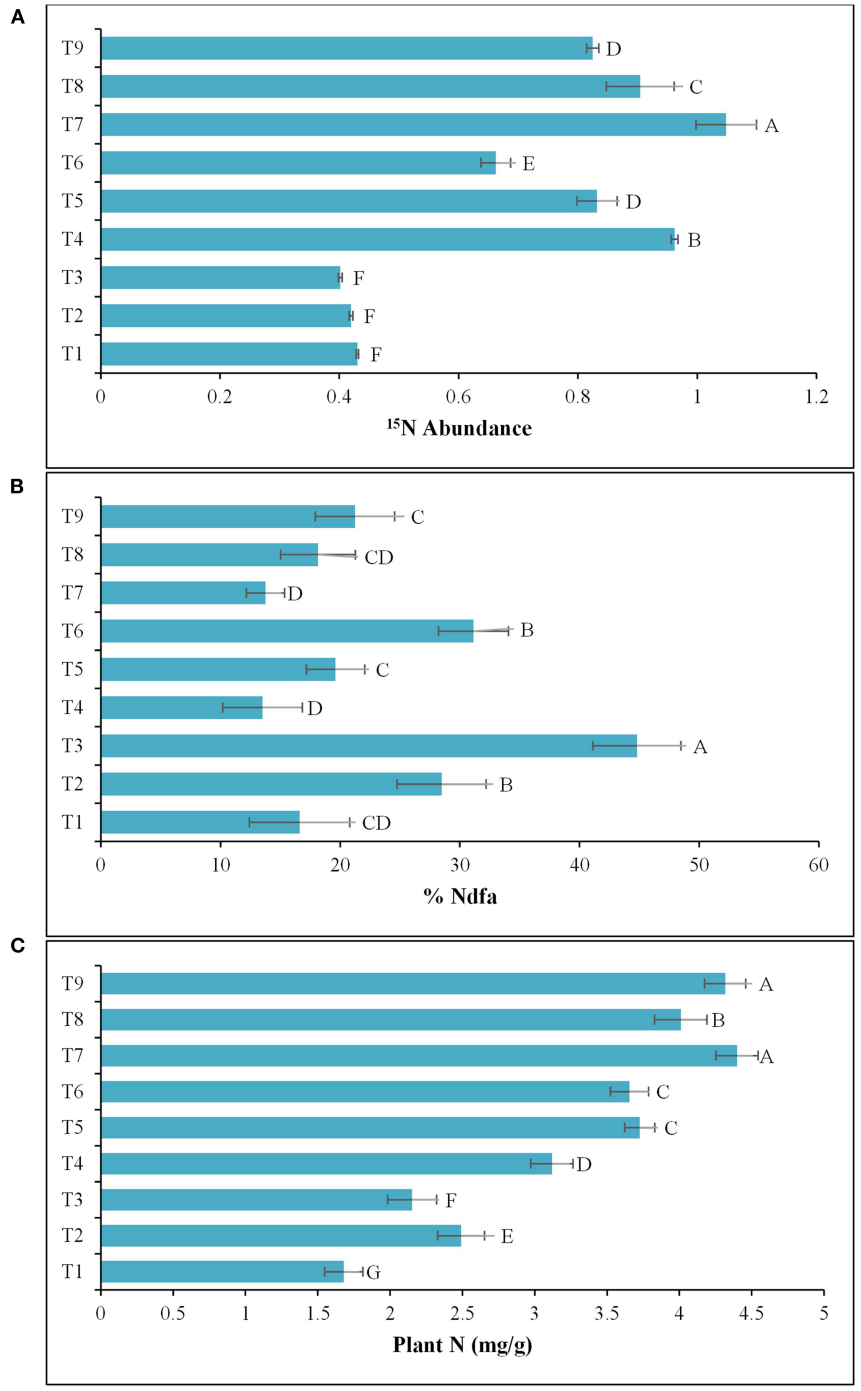
Inoculation response of *Azospirillum* spp. strains TN03 and TN09 on root and shoot: **(A)** ^15^N Abundance; **(B)** %Ndfa; and **(C)** Plant N. T_1_ = Un-inoculated + ^15^N^0^ (-ve control treatment), T_2_ = Inoculated with strain TN03 + ^15^N^0^, T_3_ = Inoculated with strain TN09 + ^15^N^0^, T_4_ = Un-inoculated + ^15^N^1/2^ (+ve control treatment), T_5_ = Inoculated with strain TN03 + ^15^N^1/2^, T_6_ = Inoculated with strain TN09 + ^15^N^1/2^, T_7_ = Un-inoculated + ^15^N^F^ (++ve control treatment), T_8_ = Inoculated with strain TN03 + ^15^N^F^, and T_9_ = Inoculated with strain TN09 + ^15^N^F^; Letters A, B, C, and D represent significant difference between means of all these treatments. Values are mean ± Standard Deviation. Number of biological replicates = 4.

The results of the 2nd pot experiment showed that the rate of N-fertilization affected the percentage of ^15^N abundance in the potato rhizosphere. The introduction of *Azospirillum* spp. (TN03 and TN09) reduced the ^15^N abundance compared compared to the respective controls (un-inoculated). The reduction in ^15^N abundance improved plant biomass and indicated the ^15^N dilution due to biological N-fixation (Figure 6A). Maximum dilution potential was observed by the introduced *Azospirillum* sp. The value of %Ndfa ranges between 13.5 and 44.8% among all the treatments (Figure 6B), indicating the effect of externally applied N-fertilizer. The highest %Ndfa value (44.8%) was recorded by *Azospirillum* sp. TN09 + N^0^, while, the minimum was observed by the *Azospirillum* sp. TN03 + N^0^ (13.5%). The reduction in ^15^N abundance also increases plant N (Figure 6C), shoot and root length (Table 3) and dry weight (Table 3), indicating the biological N-fixation potential of both isolates.

The PGP potential of both isolates *Azospirillum* spp. (TN03 and TN09) was further evaluated in field conditions at two different locations. Before conducting the experiments, the physiochemical properties and bacterial populations of both fields were investigated (Table 4). The soil samples of Faisalabad and Sahiwal fields showed sandy loamy and loamy soil texture, respectively with a slight change in total N (%) and pH. The organic matter (%) and total P (mg Kg^−1^) in the Sahiwal field were substantially higher than Faisalabad field. However, Faisalabad field soil showed an increased level of total K than Sahiwal field soil. The bacterial population in both fields ranges between 7 × 10^6^ and 7 × 10^7^ (g^−1^ soil). Introduction of *Azospirillum* spp. TN03 and TN09 in both fields yielded a positive effect on the agronomic and growth parameters of the potato plants (Supplementary Figure 4). Among the treatments in both (Faisalabad and Sahiwal) fields, the T_5_ treatment (*Azospirillum* sp. TN09 + N^1/2^) showed a significant increase in plant height (18.70 and 18.79%), plant fresh weight (22.21 and 20%) and plant dry weight (20.94 and 17.12%) than the un-inoculated negative control (Supplementary Figures 4A–D). Similarly, the inoculation of *Azospirillum* sp. TN09 + N^1/2^ in both fields remarkably increased tuber fresh weight (12.53 and 6.79%), tuber dry weight (15.59 and 13.93%), tuber yield per plot (17.91 and 21.44%) and N content of tubers (10.57 and 10.44%). However, the inoculation of *Azospirillum* sp. TN03 + N^1/2^ in both fields significantly increased the number of main branches (17.5 and 20%) and several tubers per plant (13.7 and 11.53%) than the un-inoculated negative controls (Supplementary Figures 4A–D). The numbers of compound leaves were the same in different fields under different inoculations. The introduction of *Azospirillum* sp. TN03 + N^1/2^ increased the number of compound leaves in the Faisalabad field (14.86%) while inoculation of *Azospirillum* sp. TN09 + N^1/2^ in Sahiwal field (14.41%). Moreover, the N use efficiency, nitrogen uptake, and N utilization of potato tubers in both fields were also determined (Table 5). In the Sahiwal field, maximum N utilization was observed in N^F^ treatment (1.452) and *Azospirillum* sp. TN03 + N^1/2^ (1.429) followed by *Azospirillum* sp. TN09 + N^1/2^ (1.1236), while in the Faisalabad field, *Azospirillum* sp. TN03 + N^1/2^ (1.447) maximum N utilization compared to uninoculated control potato plants. In Sahiwal field, maximum N utilization was observed in N^F^ treatment (1.452) and *Azospirillum* sp. TN03 + N^1/2^ (1.429) followed by *Azospirillum* sp. TN09 + N^1/2^ (1.1236), while in the Faisalabad field, *Azospirillum* sp. TN03 + N^1/2^ (1.447) maximum N utilization compared to uninoculated control potato plants. The N uptake in each field was same. The maximum N uptake and N use efficiency was observed in *Azospirillum* sp. TN09 + N^1/2^ in both fields followed by N^F^ and *Azospirillum* sp. TN03 + N^1/2^ treatments.

**Table 4:** Bacterial population and physiochemical characteristics of soil from Faisalabad and Sahiwal fields.

**Parameter**	**NIBGE, Faisalabad**	**PRI, Sahiwal**
Bacterial population (g^−1^ soil)	7 × 10^6^	7 × 10^7^
Soil pH	7.3	7.2
Soil texture	Sandy Loam	Loam
Total N (%)	0.06	0.08
Total K (mg Kg^−1^)	260	170
Total P (mg Kg^−1^)	53.8	64.3
Organic matter (%)	0.58	0.82

**Table 5:** Effect of *Azospirillum* inoculation and varying doses of N fertilizers on NUT, NUE and NUP.

**Field**	**Treatments**	**NUT (tones ha^**−1**^ tubers/mg g^**−1**^ N accumulated)**	**NUP (mg g^**−1**^ N accumulated/kg ha^**−1**^ N applied)**	**NUE (tones ha^**−1**^ tubers/ kg ha^**−1**^ N applied)**
Sahiwal	T1	-	-	-
	T2	0.7238 ± 0.0898	0.0118 ± 0.0018	0.0086 ± 0.0011
	T3	1.4524 ± 0.0832	0.0168 ± 0.0017	0.0244 ± 0.0010
	T4	1.4298 ± 0.0687	0.0161 ± 0.0014	0.0230 ± 0.0008
	T5	1.1236 ± 0.1099	0.0216 ± 0.0022	0.0243 ± 0.0013
Faisalabad	T1	-	-	-
	T2	1.0253 ± 0.0668	0.0118 ± 0.0013	0.0121 ± 0.0008
	T3	1.2321 ± 0.0772	0.0168 ± 0.0015	0.0207 ± 0.0009
	T4	1.4476 ± 0.0998	0.0161 ± 0.0020	0.0233 ± 0.0012
	T5	1.1633 ± 0.0907	0.0216 ± 0.0018	0.0251 ± 0.0011

**Figure 7:**
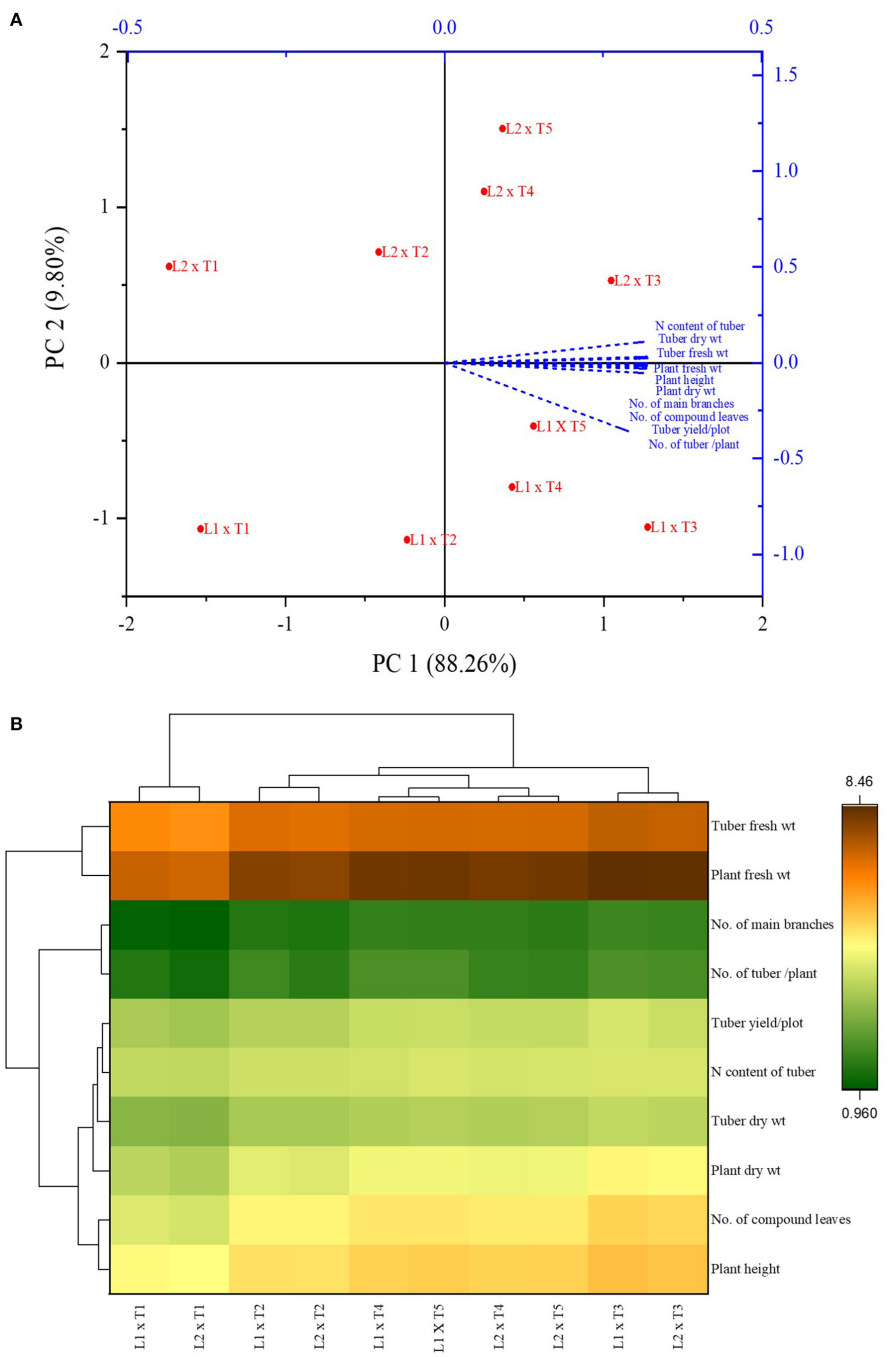
Principal component analysis **(A)** and Heatmap **(B)** of *Azospirillum* spp. strains TN03 and TN09 inoculation response on different agronomic parameters of potato plant grown in Faisalabad and Sahiwal field conditions.

PCA analysis showed the significant impact of different treatments on the growth parameters of the potato plant at two locations. All the inoculated and uninoculated treatments were differently scattered across PC1 and PC2, demonstrating their effectiveness in both locations. PC1 and PC2 accounted for 88.26 and 9.80% of the variance in data, respectively (Tables 6, 7). PC1 consisted of both inoculated treatments of both locations and showed a positive correlation with all the agronomic parameters of the potato plant (Figure 7A). However, the inoculated treatments in the Sahiwal field performed better than in the other field. The heatmap also showed that the inoculation of both *Azospirillum* sp. (TN03 and TN09) strains were positively co-related to all the growth parameters of potato plants in both field conditions (Figure 7B) and were more isolated than the other treatments, which shows better discrimination of inoculated plants from the uninoculated plants.

**Table 6:** Factor loading and values of percentage variance for different agronomic parameters obtained in PCA analysis.

**PC 1(88.26%)**	**PC 2 (9.80%)**	**Parameters**
0.31921	−0.00288	Plant height
0.32017	0.02329	Plant fresh weight
0.31936	−0.01774	Plant dry weight
0.31768	0.02483	Tuber fresh weight
0.31941	0.03222	Tuber dry weight
0.31399	0.10978	N content of tuber
0.28963	−0.35691	No. of tuber /plant
0.31825	−0.0299	No. of main branches
0.31866	−0.00942	No. of compound leaves
0.31308	−0.05349	Tuber yield/plot

**Table 7:** Factor loading and values of percentage variance for different treatments and locations obtained in PCA analysis.

**PC 1 (88.26%)**	**PC 2 (9.80%)**	**Location and treatments**
−1.53472	−1.06681	L1 × T1
−0.23639	−1.13765	L1 × T2
1.27628	−1.05653	L1 × T3
0.42356	−0.79847	L1 × T4
0.55894	−0.40664	L1 × T5
−1.73302	0.61911	L2 × T1
−0.41528	0.71182	L2 × T2
1.04765	0.52853	L2 × T3
0.24807	1.10097	L2 × T4
0.3649	1.50567	L2 × T5

## Discussion

Potato, being a vital food crop, needs substantial fertilization. To address increasing environmental concerns and the high costs of fertilizer application, this study presents the isolation and identification of two N-fixing PGP-bacteria isolated from the potato rhizosphere and demonstrates their potential for root colonization and growth promotion under field and controlled conditions. Both strains belong to the genus *Azospirillum* and phylogenetic studies validated their evolutionary relationships to *A. brasilense*. Numerous Azospirillum species have been documented for their ability to promote plant development in a variety of crops (Naqqash et al., 2016b; Zeffa et al., 2019). Both *Azospirillum* strains were metabolically active, which validates the adaptability and competency of these isolates in the rhizosphere, as metabolically diverse microbes are successful competitors and root colonizers (Wielbo et al., 2007).

Both *Azospirillum* strains showed the ability to convert acetylene into ethylene which was further confirmed by *nif* H gene amplification. The *nif* genes encode multiple subunits of the nitrogenase enzyme. Among all *nif* genes, *nif* H serves as the marker gene for investigating nitrogenase activity. Moreover, it is evolutionarily conserved and so is commonly used to identify diazotrophs (Ueda et al., 1995). Both our isolates showed *nif* H gene amplification and further analyses revealed that the sequences had 99–100% sequence similarity with the *nif* H gene sequence of *A. brasilense*. Numerous previous studies have reported the N-fixing potential of strains of *Azospirillum* in various crops (Hameed et al., 2004; Kumar and Gera, 2014; Qaisrani et al., 2014).

In the current study, both *Azospirillum* strains were capable of producing IAA. This capacity can directly promote plant growth by facilitating nutrient and water uptake. It also promotes a widening (rhizoplane) habitat for bacteria, enabling these to colonize and attach to the emerging surfaces. This promotes the microbial cycling of different nutrients, making these accessible for uptake by the plants (Majeed et al., 2015; Hakim et al., 2021). Moreover, both strains exhibited almost the same Zone for P-solubilization which is within range of the previously reported range for different PGPR (Oliveira et al., 2009; Gupta et al., 2022). Both strains also produced different organic acids which is consistent with previous studies (Shahid et al., 2012). The production of different organic acids can be linked to the P-solubilization potential of both isolates as they work together to solubilize inorganic phosphorus and reduce pH synergistically.

In the rhizospheric region, introduced bacteria need to survive, and be competitive, to exert their function. The root colonization by both strains was consistent with their survival, persistence and fitness in the potato rhizosphere. Twenty DAI, TN09 (7.21 log CFU/g) had a higher population density than TN03 (6.84 log CFU/g), however these densities declined as the crop matured. A study conducted on wheat reported that such initial population densities are sufficient to have measurable positive effects on plant growth (Fischer et al., 2010). A gradual decline in population density occurs due to developmental stages and the interactions between microbes in the rhizospheric region (Van Overbeek and Van Elsas, 2008). Further, TEM analysis demonstrated the strong associations of both isolates with plant tissue and their high numbers in the rhizosphere, potentially associated with the availability of adequate nutrients and suitable growth conditions (Guerrero-Molina et al., 2012). Confocal microscopy analyses further validated the root colonization potential of both strains. This makes both strains excellent candidates for future use as biofertilizers (Hanif et al., 2020).

Plant inoculation experiments under controlled conditions demonstrated a significant effect of inoculation on growth and N uptake in potato seedlings. Both isolates significantly increased shoot and root lengths and plant biomass compared to the uninoculated control, which can be attributable to the potential of both isolates to produce IAA and fix nitrogen. Previous studies documented beneficial effects of *A. brasilense* on growth and root development in various plants (Rondina et al., 2020; Barbosa et al., 2021). The actual contribution of introduced strains in the N supply to the potato plant was assessed using the ^15^N dilution method (Malik et al., 1987, 1991; Streeter et al., 2000; Houngnandan et al., 2008; Montañez et al., 2009a; Paungfoo-Lonhienne et al., 2014; Yonebayashi et al., 2014). The data indicated that Ndfa was greater compared to the ^15^N dilution experiment, which might be attributed to successful N-fixation by both *Azospirillum* isolates without N supplementation, however, Ndfa declined when the full recommended dose of N was administered. N is usually fixed biologically, therefore, the ^15^N in inoculated potato plants starts diluting (Malik et al., 1987). Plant biomass and total nitrogen in the potato plant were shown to be positively associated with nitrogen inputs, indicating that the potato plant benefits from both biologically fixed and supplemented nitrogen. In maize, a similar phenomenon has been reported previously (Montañez et al., 2009b). Notably, the increased root and shoot biomass acted as a diluent, as validated by the decreased concentration of total N in leaves.

Numerous studies have been conducted on potato plants to investigate the effect of PGPR (Kloepper et al., 1980; Howie and Echandi, 1983; Reitz et al., 2001; Ardanov et al., 2011; Hanif et al., 2015b; Naqqash et al., 2016a). However, such studies often were limited to controlled conditions. Clearly, PGPR may not always function properly in natural environmental conditions. Here, field studies were performed to validate the impact of both strains on potato growth and total N. In the presence of N fertilizer, diazotrophs become less responsive to nitrogen fixation (Montañez et al., 2009a), and, therefore, control treatments (N^0^, N^1/2^, and N^F^) were included to get a better understanding of the impact of the inoculants. The addition of both strains had a beneficial effect on potato biomass and total N content. The overall improvement in potato growth may not only be attributable to the IAA production and N-fixation potential of both isolates, as several other factors influence plant growth and development as described above (Reitz et al., 2001; Andreote et al., 2009; Ardanov et al., 2011; Hanif et al., 2015b; Hunziker et al., 2015). However, our results are similar to previous findings which showed that introduced *Azospirillum* can boost plant yield by 3 to 26% (Munareto et al., 2019; Caires et al., 2021). Moreover, in field conditions, *Azospirillum* isolates improved N utilization, N uptake and N use efficiency as compared to uninoculated plants, which can be due to the N fixing potential of *Azospirillum* sp. Enhanced NUE is important for the establishment of sustainable agriculture, which is now being explored on a global scale (Gaju et al., 2014).

## Conclusion

The present study demonstrates that the potato rhizosphere harbors metabolically active, N-fixing, P-solubilizing and IAA-producing *Azospirillum* species. Both *Azospirillum* sp. TN03 and TN09 possess excellent root colonization potential and increase plant biomass and N-content under both controlled and field conditions. Under field conditions, inoculation with *Azospirillum* improved N uptake, N utilization and N use efficiency of potato plants, ultimately improving plant growth. Improved N use efficiency in crops can significantly enhance farmers' economic benefits and will also reduce environmental pressure caused by excessive N fertilizer input. Thus, the use of *Azospirillum* sp. can play an important role in reducing N fertilizer inputs by increasing NUE through BNF. Thus, the here described strains can be applied as biofertilizers for improving plant growth while reducing N-fertilizer input.

## Data Availability Statement

The data presented in the study are deposited in the NCBI repository under the accession numbers: LN833443, LN833448, LT596586, and LT596588.

## Author Contributions

TN performed the experiments and wrote the paper. KAM, SH, and AI supervised, designed methodology, and experiments and improved the manuscript draft. JDvE supervised experiments. MS, AM, MJI, MKH, and MMQ validated and analyzed the data. All authors read and approved the final manuscript.

## Conflict of Interest

The authors declare that the research was conducted in the absence of any commercial or financial relationships that could be construed as a potential conflictof interest.

## Publisher's Note

All claims expressed in this article are solely those of the authors and do not necessarily represent those of their affiliated organizations, or those of the publisher, the editors and the reviewers. Any product that may be evaluated in this article, or claim that may be made by its manufacturer, is not guaranteed or endorsed by the publisher.

## Supplementary Material

Supplementary Figure 1 16S *rRNA* sequences-based phylogenetic tree of *Azospirillum* spp. strains isolated from rhizosphere of potato () and obtained from already reported sequences. The numbers at branching points represent bootstrap values > 70%. The bars show nucleotide sequence divergence of almost 0.005.

Supplementary Figure 2 *nif* H gene sequences-based phylogenetic tree of *Azospirillum* spp. strains isolated from rhizosphere of potato () and obtained from already reported sequences. The numbers at branching points represent bootstrap values > 90%. The bars show nucleotide sequence divergence of almost 0.2.

Supplementary Figure 3 Survival and population dynamics of *Azospirillum* spp. strains TN03 and TN09 in potato roots. Values are mean ± Standard Deviation. Number of biological replicates = 3.

Supplementary Figure 4 Inoculation response of *Azospirillum* spp. strains TN03 and TN09 in Faisalabad and Sahiwal fields: **(A)** Plant and Tuber Fresh weight; **(B)** Plant and Tuber dry weight; **(C)** Plant height, N content of tuber, No. of compound leaves, Tuber yield; and **(D)** No. of tuber/plant, No. of main branches. T_1_ = Un-inoculated + N^0^ (–ve control treatment), T_2_ = Un-inoculated + N^1/2^ (+ve control treatment; 175 Kg ha^−1^), T_3_ = Un-inoculated + N^F^ (+ve control treatment; 250 Kg ha^−1^), T_4_ = Inoculated with strain TN03 + N^1/2^ and T_5_ = Inoculated with strain TN09 + N^1/2^; Letters A, B, C, and D represent significant difference between means of all these treatments. Values are mean ± Standard Deviation. Number of biological replicates = 4.

Supplementary Table 1 Differential metabolic profiling of TN03 and TN09 strains associated with potato roots (Biolog PM2A Microplate analysis).
