# Supplementary figures and images for: Inoculation With Azospirillum spp. Acts as the Liming Source for Improving Growth and Nitrogen Use Efficiency of Potato
Source: Front Plant Sci. 2022 Jul 28;13:929114. doi: 10.3389/fpls.2022.929114 (PMC9366913; doi:10.3389/fpls.2022.929114)

**Supplementary Figure 1**

**
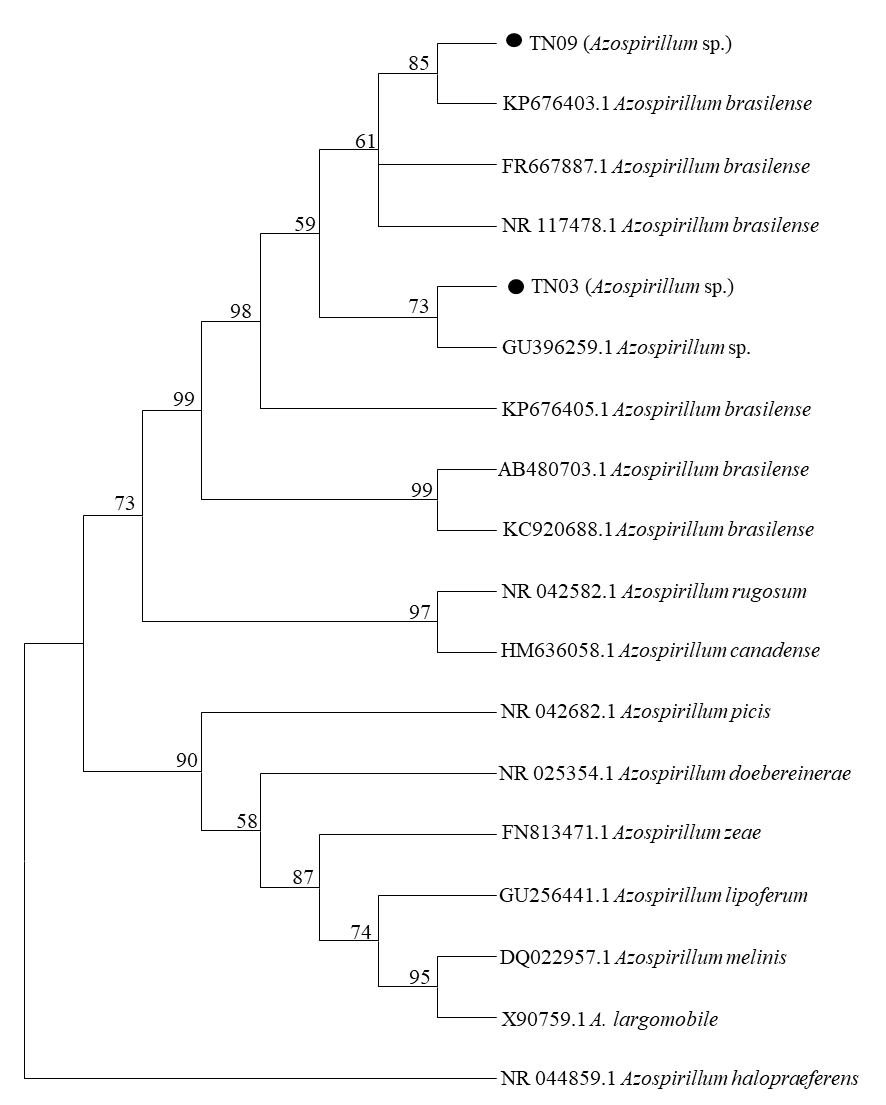
**

**Supplementary Figure 2**
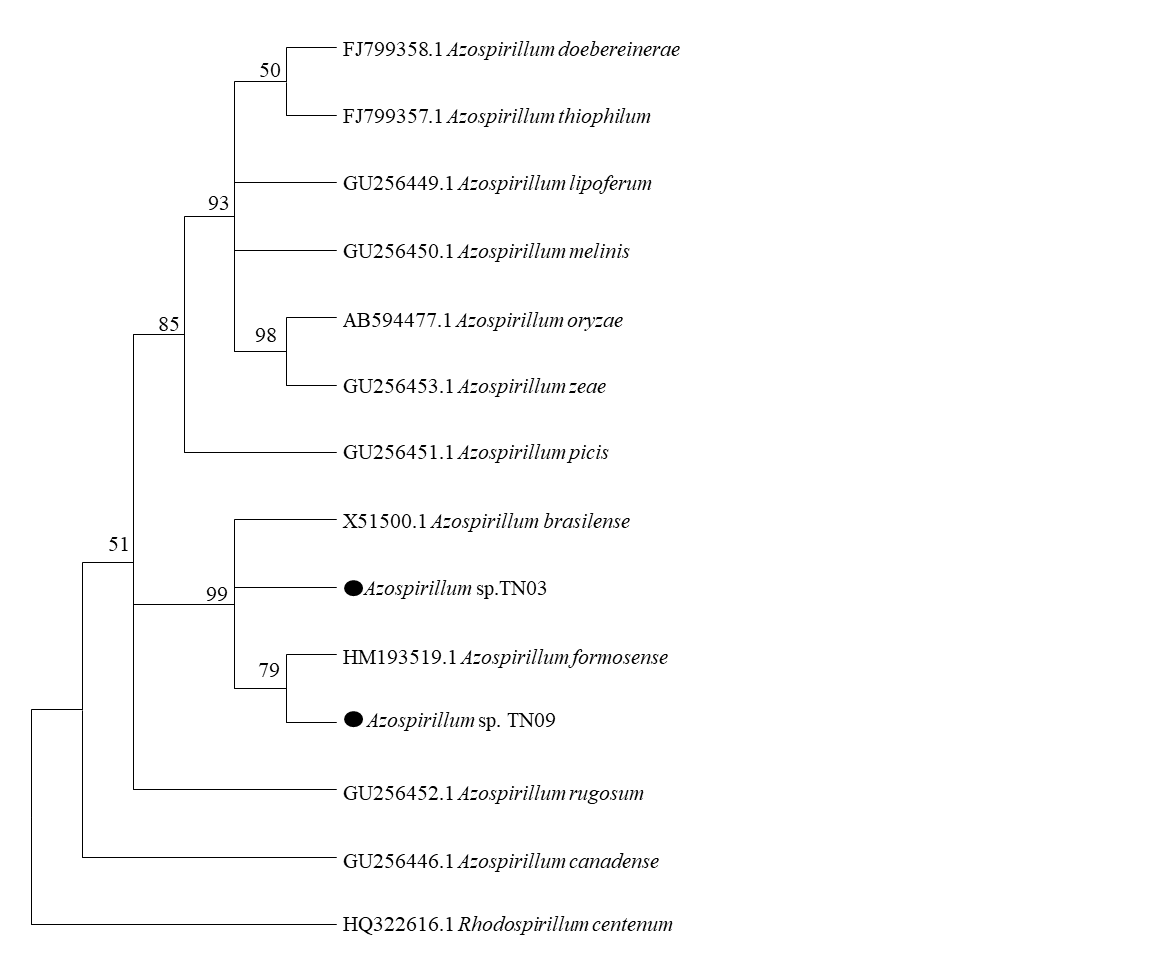


**Supplementary figure 3**

**Supplementary Figure 4**

**
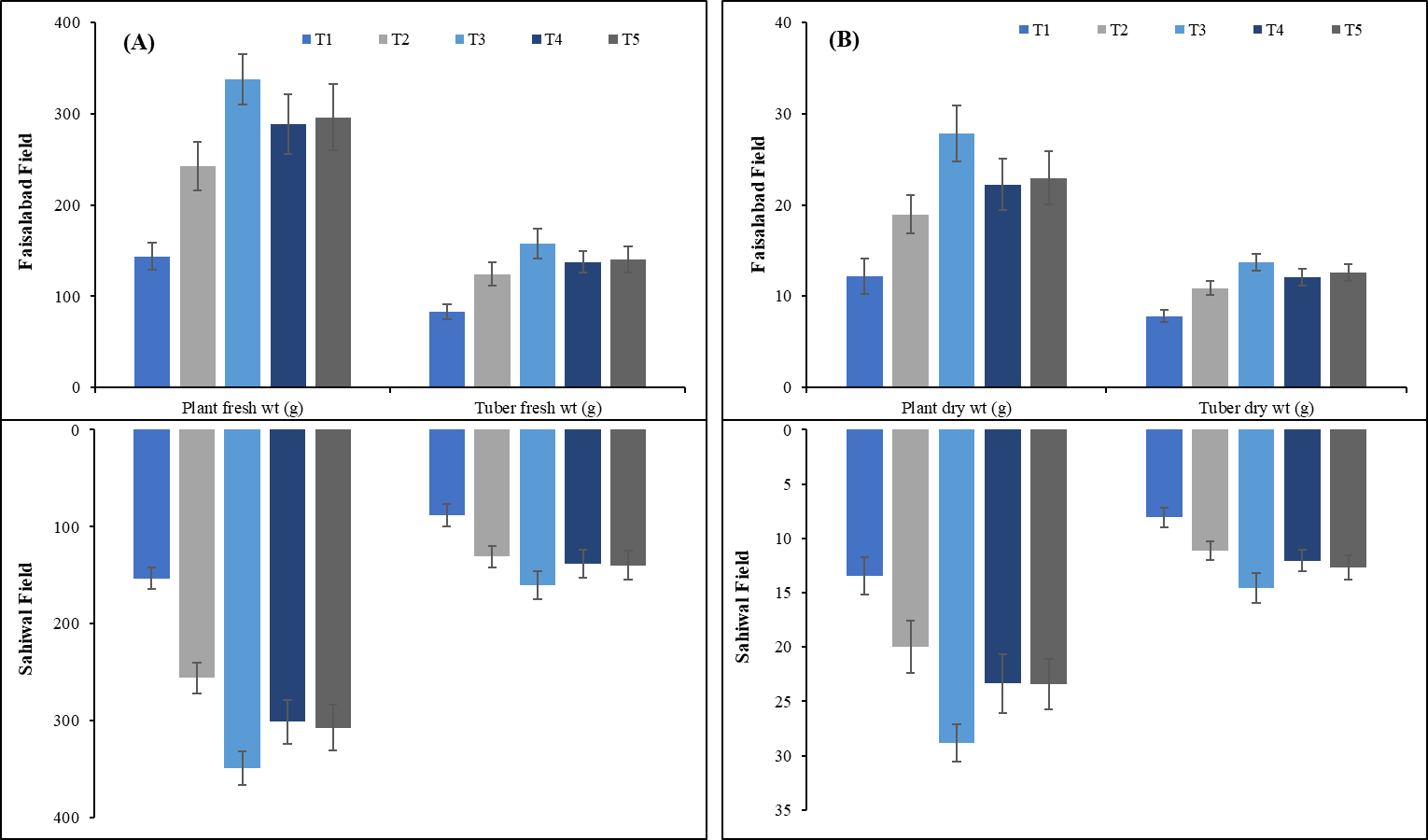

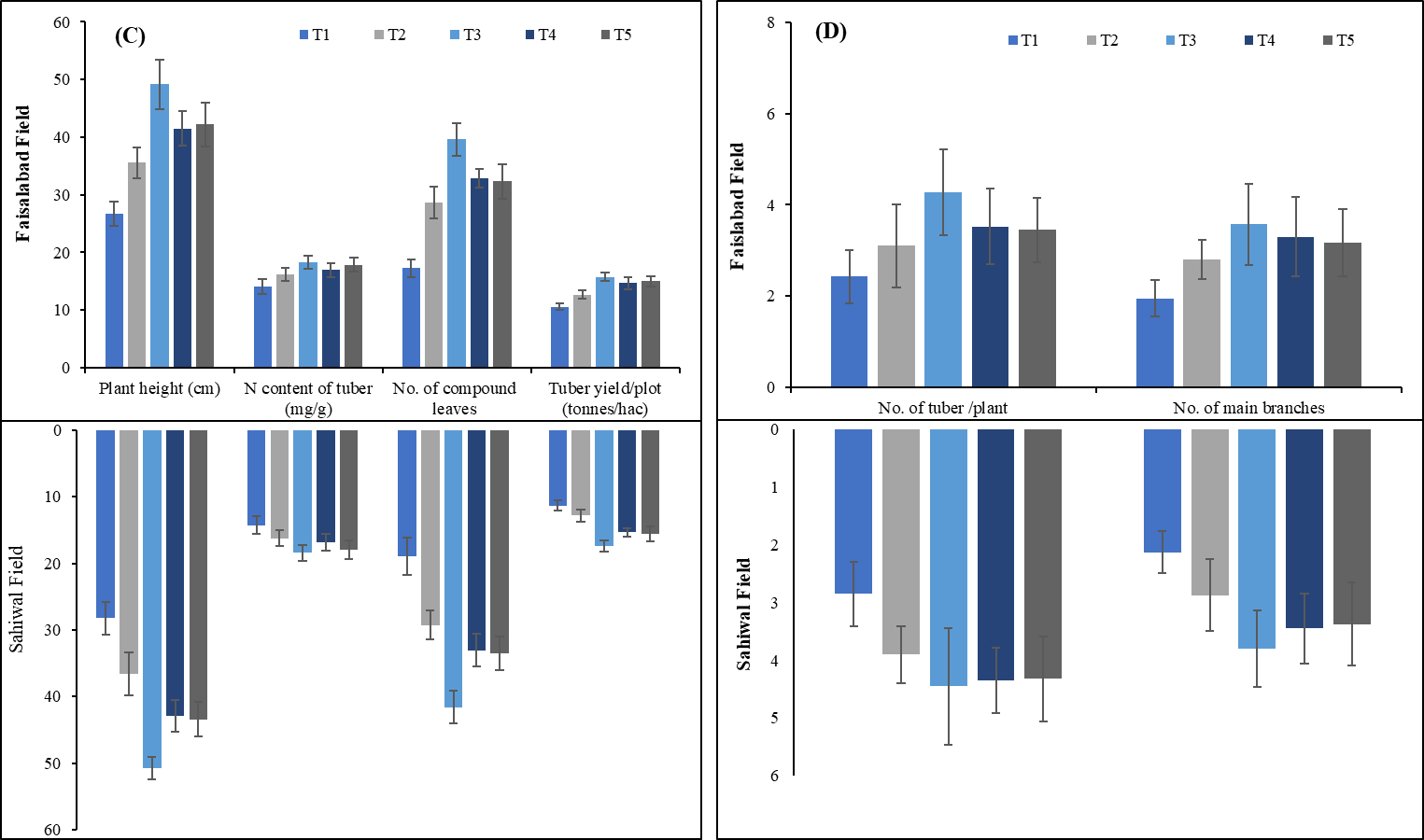
**

Supplement: Supplementary Figure 1 — 16S rRNA sequences-based phylogenetic tree of Azospirillum spp. strains isolated from rhizosphere of potato () and obtained from already reported sequences. The numbers at branching points represent bootstrap values > 70%. The bars show nucleotide sequence divergence of almost 0.005. [file Data_Sheet_1.docx]
