# Supplementary material for: Inoculation With Azospirillum spp. Acts as the Liming Source for Improving Growth and Nitrogen Use Efficiency of Potato
Source: Front Plant Sci. 2022 Jul 28;13:929114. doi: 10.3389/fpls.2022.929114 (PMC9366913; doi:10.3389/fpls.2022.929114)
Supplement: Supplementary Table 1 — Differential metabolic profiling of TN03 and TN09 strains associated with potato roots (Biolog PM2A Microplate analysis). [file Table_1.docx]

**Supplementary Table 1 Differential metabolic profiling of TN03 and TN09 strains associated with potato roots (Biolog PM2A Microplate analysis)**

| **Carbon Source** | **TN03** | **TN09** | **Carbon Source** | **TN03** | **TN09** |
| --- | --- | --- | --- | --- | --- |
| **Chondroitin Sulfate C** | **-** | **-** | **D-Melezitose** | **-** | **-** |
| **α-Cyclodextrin** | **-** | **-** | **Maltitol** | **-** | **-** |
| **β-Cyclodextrin** | **-** | **-** | **α-Methyl-D-Glucoside** | **-** | **-** |
| **γ-Cyclodextrin** | **-** | **-** | **β-Methyl-D-Galactoside** | **-** | **-** |
| **Dextrin** | **+** | **-** | **3-Methyl Glucose** | **-** | **-** |
| **Gelatin** | **-** | **-** | **β-Methyl-D-Glucuronic Acid** | **-** | **-** |
| **Glycogen** | **+** | **-** | **α-Methyl-D-Mannoside** | **-** | **-** |
| **Inulin** | **-** | **-** | **β-Methyl-D-Xyloside** | **-** | **-** |
| **Laminarin** | **-** | **-** | **Palatinose** | **-** | **-** |
| **Mannan** | **+** | **+** | **D-Raffinose** | **-** | **+** |
| **Pectin** | **-** | **-** | **Salicin** | **+** | **-** |
| **N-Acetyl-D-Galactosamine** | **-** | **-** | **Sedoheptulosan** | **-** | **-** |
| **N-Acetyl-Neuraminic Acid** | **-** | **-** | **L-Sorbose** | **+** | **+** |
| **β-D-Allose** | **-** | **-** | **Stachyose** | **-** | **-** |
| **Amygdalin** | **-** | **-** | **D-Tagatose** | **-** | **-** |
| **D-Arabinose** | **-** | **-** | **Turanose** | **-** | **-** |
| **D-Arabitol** | **-** | **-** | **Xylitol** | **-** | **-** |
| **L-Arabitol** | **-** | **-** | **N-Acetyl-D-Glucosaminitol** | **-** | **-** |
| **Arbutin** | **+** | **+** | **γ-Amino Butyric Acid** | **+** | **-** |
| **2-Deoxy-D-Ribose** | **-** | **-** | **δ-Amino Valeric Acid** | **-** | **-** |
| **i-Erythritol** | **-** | **-** | **Butyric Acid** | **-** | **+** |
| **D-Fucose** | **+** | **+** | **Capric Acid** | **-** | **-** |
| **3-0-β-D-Galacto-pyranosyl-D-Arabinose** | **-** | **-** | **Caproic Acid** | **-** | **-** |
| **Gentiobiose** | **-** | **-** | **Citraconic Acid** | **-** | **-** |
| **L-Glucose** | **-** | **-** | **Citramalic Acid** | **-** | **-** |
| **Lactitol** | **-** | **-** | **D-Glucosamine** | **+** | **-** |
| **2-Hydroxy Benzoic Acid** | **-** | **-** | **L-Arginine** | **-** | **-** |
| **4-Hydroxy Benzoic Acid** | **-** | **-** | **Glycine** | **-** | **-** |
| **β-Hydroxy Butyric Acid** | **-** | **-** | **L-Histidine** | **-** | **-** |
| **γ-Hydroxy Butyric Acid** | **-** | **-** | **L-Homoserine** | **-** | **-** |
| **α-Keto-Valeric Acid** | **-** | **-** | **Hydroxy-L-Proline** | **-** | **-** |
| **Itaconic Acid** | **-** | **-** | **L-Isoleucine** | **-** | **-** |
| **5-Keto-D-Gluconic Acid** | **-** | **+** | **L-Leucine** | **-** | **-** |
| **D-Lactic Acid Methyl Ester** | **-** | **-** | **L-Lysine** | **-** | **-** |
| **Malonic Acid** | **-** | **-** | **L-Methionine** | **-** | **-** |
| **Melibionic Acid** | **-** | **+** | **L-Ornithine** | **-** | **-** |
| **Oxalic Acid** | **-** | **-** | **L-Phenylalanine** | **-** | **-** |
| **Oxalomalic Acid** | **-** | **-** | **L-Pyroglutamic Acid** | **-** | **-** |
| **Quinic Acid** | **-** | **-** | **L-Valine** | **-** | **-** |
| **D-Ribono-1,4-Lactone** | **-** | **-** | **D,L-Carnitine** | **-** | **-** |
| **Sebacic Acid** | **-** | **-** | **Sec-Butylamine** | **-** | **-** |
| **Sorbic Acid** | **-** | **-** | **D.L-Octopamine** | **-** | **-** |
| **Succinamic Acid** | **-** | **-** | **Putrescine** | **-** | **+** |
| **D-Tartaric Acid** | **-** | **-** | **Dihydroxy Acetone** | **-** | **-** |
| **L-Tartaric Acid** | **-** | **-** | **2,3-Butanediol** | **-** | **-** |
| **L-Alaninamide** | **-** | **+** | **2,3-Butanone** | **-** | **-** |
| **N-Acetyl-L-Glutamic Acid** | **-** | **-** | **3-Hydroxy 2-Butanone** | **-** | **-** |
